# Evidence-based preschool-age vision screening: health policy considerations

**DOI:** 10.1186/s13584-019-0339-z

**Published:** 2019-09-12

**Authors:** Deena Rachel Zimmerman, Hadas Ben-Eli, Bruce Moore, Monique Toledano, Chen Stein-Zamir, Ariela Gordon-Shaag

**Affiliations:** 1 Jerusalem District Health Office, Ministry of Health, Jerusalem, Israel; 2 Department of Optometry, Hadassah Academic College, Jerusalem, Israel; 3 New England College of Optometry, Boston, MA USA; 4 The Hebrew University of Jerusalem, Faculty of Medicine, The Hebrew University and Hadassah Braun School of Public and Community Medicine, Jerusalem, Israel; 5 Department of Ophthalmology, Hadassah-Hebrew University Medical Center, Jerusalem, Israel

**Keywords:** Vision screening, Preschool, Amblyopia, Strabismus

## Abstract

**Background:**

There are many causes of visual impairment, and even blindness, which are treatable or at least preventable. Two such conditions are strabismus (crossed-eye, squint) and refractive error (visual image not focused on the most sensitive part of the retina). If these are not detected and corrected at an early age, they can lead to an irreversible impairment known as amblyopia (lazy eye). Pediatric vision screening and subsequent treatment for amblyopia and amblyogenic risk factors are thus key to preventing vision loss. Furthermore, vision screening can detect moderate to high hyperopia, which has been found to be associated with poor school readiness.

Evidence-based recommendations call for screening children at 3–5 years of age; they are old enough to cooperate, but still within the window of effective intervention. However, these recommendations have yet to be universally implemented as the standard of care.

**Methods:**

This paper integrates a review of the literature and the international experience of preschool vision screening with the findings from a preliminary feasibility study of expanded screening in Israel to formulate a discussion of the current health policy challenge in Israel and the options for addressing it. The advantages and disadvantages of various venues for vision screening are discussed.

**Findings:**

Screening by optometrists in Mother and Child Health Centers, as implemented in a recent pilot project in the Jerusalem District, would allow the most comprehensive testing. Photo-screening in preschools would reach the most children, but at the cost of missing hyperopia (farsightedness). Either approach would probably constitute improvements over the current situation. The relative strengths of the two approaches depends in part on the ability to purchase automatic screening equipment (and the efficacy of that equipment) vs. the ongoing cost of paying trained personnel.

**Conclusions:**

Further research should be conducted in Israel to determine the prevalence of refractive errors, so that best practices can be established for Israel’s population and social needs. In the interim, the Ministry of Health should promptly implement the inclusion of preschool visions screening for children in the approved “basket of services” covered by the National Health Insurance Laws, using photo-screening, including collection of the clinical data.

**Electronic supplementary material:**

The online version of this article (10.1186/s13584-019-0339-z) contains supplementary material, which is available to authorized users.

Vision makes a crucial contribution to optimal child development and lifelong functioning.

Preschool screening can uncover conditions that impair future vision. Currently, many children in Israel in this age group are not screened. The objective of this paper is to present the challenges regarding vision screening in Israel via 1) a literature review of local and international screening practices 2) discussion in light of the findings of a preliminary feasibility study and 3) potential health policy changes.

## Background

Vision makes a crucial contribution to childhood development, education, employability and lifelong independence [1]. Vision impairment places a financial burden on the economy and significantly contributes to poverty [2]. A 2010 review showed that 65% of blind people and 76% of visually impaired people (worldwide) had a preventable or treatable condition. Many cases were caused by uncorrected refractive error, even in developed countries [3, 4] [5]. A refractive error occurs when the eye cannot clearly focus the images from the outside world. The result of refractive errors is blurred vision, which is sometimes so severe that it causes significant visual impairment. For example, the percentage of avoidable blindness from uncorrected refractive error is 14% in Western Europe, 41% in South Asia and 23% in the Middle East. In preschool children in the United States, it has been estimated that the number one cause of vision impairment is uncorrected refractive error [6]. Moreover, uncorrected refractive error in children can lead to the more worrisome sequela of amblyopia.

### Amblyopia

Amblyopia is the potentially permanent reduction of vision in one or both eyes caused by conditions that adversely affect the normal development of vision [7]. Any condition that does not allow equal stimulation of both eyes leads to structural and functional abnormalities of the visual cortex [7]. These conditions include: strabismus, in which the eyes are crossed inward (esotropia) or turned outward (exotropia), significant unilateral or bilateral refractive error and anisometropia (a major difference in refractive error between the two eyes). Intervention at a young age can restore relatively normal sight, with children younger than seven years of age having the best outcome [8].

The prevalence of amblyopia and strabismus in preschool children in the United States has been investigated in several large prospective multi-ethnic studies [9–11]. They demonstrated a prevalence of amblyopia of 3–5.4% and a prevalence of strabismus at 1.0–4.6% [9–11]. An ethnic difference was observed although the results were not statistically significant.

In Israel, the prevalence of amblyopia has not been addressed in preschool children but has been reviewed retrospectively in military pre-recruits [12, 13] and prospectively in a small sample of 8-year old children [14] (Table 1). The studies in pre-recruits may not reflect the accurate prevalence of amblyopia since the cohort does not include Israeli Arabs and teenagers with disabilities, both of whom are exempt from army service, nor those children who had conditions that would have caused amblyopia but were treated in childhood. The prospective study of 8-year old children found amblyopia to be prevalent in 1% of children in the Haifa district who were screened as infants and in 2.6% in an unscreened cohort in the nearby Hadera district [14]. A separate study looking at the prevalence of strabismus in schoolchildren in Northern Israel found rates to be 1.2–1.4% and 0.3–1.0% for first graders and eighth graders, respectively. [15] However, that study did not assess the prevalence of amblyopia.

**Table 1:** Prevalence of Amblyopia in Israel

Study	Type	Population	Rate of amblyopia
Shapiro et al. 2017 [13]	Retrospective	107,608 pre-enlistees 17.5 0.6 years age. Northern Israel born 1971–1994	Current 0.8% from 1.2%
Ore et al. 2009 & 2014 [15, 16]	Prospective	2113 first and eighth grade schoolchildren	Did not measure amblyopia. Strabismus found to be 1.2–1.4% and 0.3–1.0% for 1st and 8th graders, respectively
Morad et al. 2007 [12]	Retrospective	305,712 pre-enlistees 17.5 0.6 years age. Entire country 1981–1986	Native born Israelis 0.98, immigrants 1.5%
Eibschitz-Tsimhoni et al. 2000 [14]	Prospective	8 year olds born ~ 1986 808 Haifa -pre-screened 782 Hadera – no screening	Haifa prescreened 1% Hadera – no screening 2.6%

### Vision screening to reduce amblyopia

A longitudinal study in Sweden offers evidence that pediatric vision screening can greatly reduce amblyopia. In Sweden, preschool screening and subsequent diagnosis and treatment reduced the prevalence of amblyopia from 2 to 0.2% [17].

No studies conducted in Israel to date provide direct evidence that vision screening prevents amblyopia. However, indirect evidence suggests that this is the case. Shapiro hypothesizes that the reduction in amblyopia over 25 years in Israel is a result of pediatric screening program, although he does not clarify the nature of that screening program [13]. Furthermore, Morad postulates that the lower rate of amblyopia found in native Israelis is due to better screening in Israel than in the former Soviet Union [12]. While these investigations do not show a causative benefit from vision screening (rather an association), we assume, based on international experiences, that this would be the case in Israel as well.

### Additional benefits of vision screening

Aside from amblyopia prevention, an additional benefit of pediatric vision screening is detection of children with moderate to high hyperopia (farsightedness) and correction with glasses. Hyperopia is associated with decreased visuo-cognitive ability, reading ability, and visual attention in young children [18–31]. The prevalence of moderate hyperopia depends on ethnicity, with the lowest rate in Asians (5.5%) and the highest in non-Hispanic whites (11.9%) [32]. The prevalence of hyperopia (of any degree) has been assessed in Israeli first graders in Northern Israel and found to be 13.1 and 7.1% for Arabs and Jews, respectively (*p* < 0.003) [15]. However, the authors did not define the nature of this hyperopia so it is not possible to determine the prevalence of moderate to high hyperopia.

The association between hyperopia and impaired reading ability is thought to begin in preschool [29]. Two recent studies [33, 34] imply that some preschool children with uncorrected hyperopia start school with an educational deficit in early literacy, attention, visual-motor integration and visual perception. The corollary is that early detection and spectacle correction of hyperopia in preschool may allow children to catch up and start first grade without an educational deficit [26].

There is broad consensus as to the benefit of pediatric vision screening. However, a number of issues are not yet fully resolved, including the recommended age for screening and the recommended process for screening; we will now discuss those two issues in turn.

### Recommended age for screening

Vision screening is recommended for infants by several professional organizations. For example, the American Academy of Ophthalmology recommends screening [35] from 6 months to a year while the American Association for Pediatric Ophthalmology and Strabismus recommends screening at least once from one year to 36 months [36]. However, review of this approach by United States Preventive Services Task Force (USPSTF), an independent panel of experts in evidence-based preventive medicine, did not find support in the literature for the risk-benefit balance of screening at this age. They concluded that the current evidence is insufficient to assess the net benefit of screening children younger than three, and that there are no proven effective screening techniques for this age. In addition, Children younger than three are often unable to cooperate with testing and either yield false-positive results or do not complete a full screening. Therefore, the USPSTF determined that 3–5 years is the optimal age to perform vision screening [37, 38] and recommends screening all children aged 3 to 5 years at least once to detect amblyopia and its risk factors, with yearly screening a better option. While ages six and older are most likely to cooperate, screening and initiating therapy at this later age may preclude optimal results as children younger than seven years are most responsive to amblyopia treatment [39, 40].

### Recommended procedures for screening

The Vision in Preschoolers Study group (VIP) has published extensively on the evidence for the efficacy of various screening procedures [41–53] . In addition, the National Expert Panel to the National Center for Children’s Vision Eye and Health have published evidence-based recommended practices [54, 55]. The sensitivity and specificity vary by technique, person performing the procedure (professional vs. layperson), and prevalence of risk factors in the given population [56]. In addition, the fail criteria for each procedure differ between studies. The following section will describe the results of those procedures tested by the VIP study group that show the highest sensitivity, with specificity set at 90% and using the American Academy of Pediatric Ophthalmology and Strabismus failure criteria.

The best screening technique to detect children with the highest risk for amblyopia is by non-cycloplegic retinoscopy performed by an ophthalmologist or optometrist trained to perform this technique on children [47]. Retinoscopy involves shining a beam of light into the eye and measuring objectively refractive error based on the reflection of the light off the retina. Use of automated refractive photo-screeners in the hands of non-eye care professionals is the next best technique after retinoscopy [57]. Each method has advantages and disadvantages; while retinoscopy requires ongoing expensive human resources, photo-screening requires an initial expensive capital investment in equipment and misses some children with moderate to high degrees of hyperopia [58].

Distance visual acuity is currently the most commonly performed pediatric vision screening test. However, children with moderate hyperopia and astigmatism can sometimes perform satisfactorily on a visual acuity test and thus be missed. Furthermore, if the screening environment and the screener’s technique do not follow protocol, it can result in significant deficits in accuracy [55].

Binocular vision examinations such as the cover test, the near point of convergence tests, and various versions of stereopsis tests can be more effective in detecting subtle amblyopia and strabismus compared to refractive or visual acuity screening tests [49]. Their use can therefore improve the accuracy of the vision screening. Moreover, indirect evidence supports the benefit of using multiple screening tests for identifying preschool children at higher risk for vision problems [56]. However, binocular vision examinations also suffer from the same limitations as visual acuity testing. Furthermore, they require training to be performed and interpreted correctly, which can add to the cost of screening.

### Vision screening around the world

Different vision screening services are provided around the world. In particular, various national organizations in the United States have issued guidelines for vision screening of children from birth through childhood. The National Center for Children’s Vision and Eye Health [55] published a detailed assessment of evidenced-based techniques and a model data collection systems [54]. The American Academy of Pediatrics [59] incorporated a highly detailed program of all screening recommendations into its well child care guidelines for pediatricians known as Bright Futures [60]. A joint committee of the American Academy of Pediatrics, the American Academy of Ophthalmology, and the American Association of Pediatric Ophthalmology and Strabismus [36] recommended specific procedures (mostly automated refracting devices and visual acuity testing) at appropriate ages and intervals throughout early childhood.

However, the United States is comprised of 50 individual states with differing laws and health care systems, and thus there is enormous variability across the country. Some of these systems work well in providing high quality vision screening, follow-up, and treatment of vision problems, others much less so.

In Europe and neighboring countries, at least 35 countries have some sort of national program funded by the state, region or insurance provider [61]. Visual acuity testing is performed in all screening programs, with first screening at 3–7 years of age, depending on the country. Screening is mostly performed by physicians, ophthalmologists or nurses.

### Status of vision screening in Israel

Based on recommendations of the Israel Neonatal Society, all newborns in Israel are tested for the presence of a red-reflex prior to discharge from hospital, with the hope of identifying the rare child with retinoblastoma, congenital cataract or gross anomalies of the eye and ocular adnexa [62]. Visual acuity screening, performed by nurses at ages 3 and 5, are part of the national preventive health services offered at community based Maternal Child Health Clinics (MCHC). However, the visual acuity charts that are currently used have been shown to be unacceptable for this age cohort [63]. Furthermore, distance screening alone does not test for strabismus, astigmatism and hyperopia and may miss amblyopia. An additional problem is that compliance with clinic visits at this age is very low in many areas of the country. Distance visual acuity screening in first grade is part of the school health services funded by the national government. However, these children are close to the end of the optimal age of prevention of amblyopia and distance visual acuity may miss children with strabismus, hyperopia and astigmatism.

There are additional interventions without national coordination. The municipality of Jerusalem had subcontracted a local hospital to provide vision screening for nine months old babies, but this service has been discontinued. One of the health funds that are part of Israel’s universal health care, reminds physicians to refer all children under age one to an ophthalmologist. However, this is not a practical or realistic use of resources, as there are only 55 pediatric ophthalmologists listed in all four health funds and approximately 700,000 children in Israel between ages 3–6. Use of such limited resources is against the principles of screening: screening programs are generally designed to be a multistage process whose first step is to identify those at high risk and then send this group for definitive diagnosis [64].

The first step to finding the ideal screening procedure in Israel is to implement an evidence-based protocol based on best practices from other countries and test its feasibility. To this end, the Jerusalem District Health Office, in cooperation with Hadassah Academic College Department of Optometry (HACO), piloted a screening program based on the VIP evidence-based protocol at MCHC [65]. Simultaneously, HACO implemented the same screening program at preschools in the Jerusalem area. Study methodology and results for both can be found in the Additional file 1.

The overall referral rate for this preliminary feasibility project was 24%, with a higher rate at the preschools (26%) than at the MCHC (21%), but not significantly so. That said, the MCHC referral rates trended closer to the range previously reported in the literature of 10–20% [43] There are no current data on failure of preschool vision testing in Israel. The closest data comes from a study by Ore et al., which found a prevalence of visual acuity worse than 6/12 in 29 and 15% of Arab and Jewish first graders, respectively. These first graders would have been referred according to the criteria used in our study, resulting in a similar failure rate. Comparing referral rates between studies is difficult, however, due to differences in population types and screening technique.

The finding of our study show that in Israel, similar to other locations, 24% of children are at risk of vision difficulties. Therefore, in concert with policy decisions in other countries, it would appear that a national program of preschool vision screening should be considered in Israel. The pilot described in the Additional file 1 showed that using optometry students supervised by a licensed optometrist is one feasible screening option. Almost all children cooperated with the exam. In the MCHC, where parents were present, almost all families reported to the staff that they were happy with the thoroughness of the examination. A benefit of this venue (as opposed to the preschool setting) was parental presence as it was possible to give parents direct instructions about the need for follow-up. In addition, the public health nurse at MCHC received the results and was responsible for reminding parents of the need for follow up, which is an essential element of a vision screening program [54]. The main limitation of our pilot study is that no follow-up was done to determine the sensitivity and specificity of the screening and the degree of parental compliance in assuring that those children who were referred in fact had further assessment. Such a follow up study is currently being conducted.

This pilot could not be implemented on a nationwide scale as part of the clinical training of optometry students; there are only two schools of optometry and they are located in Jerusalem and Ramat Gan, making it difficult to access many of the children in the country. Rather, licensed optometrists would need to be recruited to perform this service. According to the Israel Central Bureau of Statistics there were ~ 183,648 births in 2017 [66]. Expert optometrists could screen four children per hour seven hours a day (allowing for breaks). Given an average of 247 workdays a year, ~ 27 full time optometrists would be required to screen an entire birth year cohort. Whether the optometrist would be employed by the Ministry of Health or work as subcontractors remains to be determined. Other costs would include equipment, logistic and administrative support and a consulting ophthalmologist.

The difficulty with the MCHC screening was attendance. Only 25% of parents who were contacted scheduled an appointment and brought their children in for the screening procedure. It is likely that if there were more scheduling flexibility, particularly by making afternoon hours available, the compliance would be better.

Another possibility would be to perform the vision screening on younger babies when they present for immunization at an MCHC. This would likely yield a higher coverage since compliance with MCHC visits at those ages is very high. However, based on studies from the United States, the evidence is insufficient to assess the benefits of vision screening in children younger than three years [38, 56, 67]. A large-scale screening study must be carried out in Israel to determine the benefits of screening younger babies in our population. The screening study in Haifa [14] provides evidence that screening young babies indeed lowers the prevalence of amblyopia, but we believe that a larger study is needed to make sure there is enough evidence to screen at a younger age. Younger ages are also often more time consuming in obtaining the child’s cooperation.

In the pilot study, screening coverage rates were better at the preschool location due to having a “captive audience” without the need to invite children to participate. This captive audience approach is planned as the basis of the vision screening that was approved to start as part of the “basket of services” covered by Israel’s National Health Insurance Law. It is planned that children will be screened in the preschool setting using photo-screeners. This is likely to achieve good screening coverage of this age cohort. However, it is not clear, if the letter indicating the need for follow-up will reach the parents and if they will understand the implication of the referral for comprehensive vision testing. Moreover, in the preschool setting there is no public health worker responsible for reminding the parents to comply with the referral. Furthermore, these instruments have been shown to miss children with moderate to high hyperopia.

## Policy implications

Based on the results of this pilot and literature describing the international experience described above, further research should be conducted in Israel to obtain local data so that best practices can be established for our population and social needs.

Several steps are needed to reach this goal. The first is to determine normative data and epidemiology of vision issues, (VA at various ages, refractive error at various ages, and binocularity) in the different populations in Israel. While findings from other countries such as the USA are a good starting point, normative data is often specific to a given ethnicity and environmental setting. Thus, we suggest carrying out these experiments on various ethnic and social groups in Israel. In Israel, we may find different epidemiology patterns for refractive error in Jewish, Arab and ultra-Orthodox Jewish populations [16, 68].

The second goal should be to develop a consensus by the Israeli eye care community of where referral cutoffs should be set, using USA -based or other data as a starting point, but interpreted in light of the Israeli healthcare system and visual needs. Since the healthcare system in Israel is vastly different from the USA, where many of the studies were performed, this must be adapted. For example, the ratio of ophthalmologists and optometrist and their scope of practice in Israel is different. While Israel and the USA have a similar number of ophthalmologists per population [69] there is a vast gap regarding optometrists due to the limited scope of optometry practice in Israel. In the USA, the scope of optometry practice includes the use of diagnostic pharmaceuticals, allowing them to treat children and provide primary eye care. This is not the case for Israel, leaving Israel with a deficit in eye care professionals who can provide primary care. Furthermore, all Israel’s children are covered by health insurance which is not the case at present in the USA.

The next step would be to carry out a study similar to the Vision in Preschool Study to compare and validate screening methods according to the newly established cutoffs for each method of screening. Again, the VIP study would be a good starting place. However, the referral cutoffs and visual needs might be different based on the Israel healthcare system and visual needs in society. For example, many states in the USA require good vision in both eyes, while in Israel people with amblyopia do not have driving restrictions if they have one good eye.

At the end of these steps and based on the results, Israel would have to determine the best venue for vision screening. There are several options: MCHC, preschools, pediatricians at the healthcare funds and optometrists at healthcare funds. The advantages and disadvantages of each venue are described in the following table.

In the interim, a vision screening program done in the preschool educational setting by photo-screening would be a good first step. This service entered the basket of services in this past year and its implementation is being planned. This will at least assure national focus on this important topic and screen a large number of children in a “captive” setting. However, the limitations of this approach are as described in Table 2 and the lack of national referral standards means that continued study as outlined above, is still needed.

**Table 2:** Comparison of different venues for preschool vision screening

Location	Advantages	disadvantages
MCHC	1.Location of well-child pediatric visits in Israel 2.Presence of parents 3. Built in follow-up	1. Staff overwhelmed with current workload. 2. Low compliance with well-child screening at relevant ages. 3. Sensitivity lower when pediatricians and nurses do vision screening techniques such as VA, cover-test, red reflex, motilities, etc., 4.Intensive training would be needed for pediatricians and nurses to use retinoscopy 5. Optometrists performing vision screening would require addition human resources and accompanying expense.
Preschool	Can get high coverage due to “captive audience”	1.No follow-up built into the system 2. Parents not present. 3.School health services are not currently uniform – some government, some private 4.Staff overwhelmed with current responsibilities so new staff would have to be hired or service out sourced
Pediatrician at healthcare funds	High coverage since most children of relevant ages see a pediatrician at least once during relevant time period	1. These are primarily sick-child visits. Screening a sick child can give invalid results. 2. Time constraints of pediatricians' acute care visits. 3. Same limitations about types of exams as discussed for MCHC. 4. Standard well child visits was first recommended in 2019 [70]. Compliance for well visits likely to be low in near future.
Optometrist at healthcare funds	1.Trained professionals 2. Good controlled environment for screening children. 3.Parents present 4. Data would be part of electronic medical record and thus facilitate follow-up	1.Currently unfunded 2. Parental compliance for any well visit likely to be low.

In conclusion, a comprehensive approach to the development of a national vision screening programs for preschool children is needed. There are advantages and disadvantages of various venues, professionals and screening tests. Any of the discussed approaches would be an improvement over the current situation.

## Additional file

Additional file 1: Appendix 1. (DOCX 20 kb)

## Data Availability

The datasets during and/or analyzed during the current study available from the corresponding author on reasonable request.
