# Supplementary material for: Evidence-based preschool-age vision screening: health policy considerations
Source: Isr J Health Policy Res. 2019 Sep 12;8:70. doi: 10.1186/s13584-019-0339-z (PMC6739935; doi:10.1186/s13584-019-0339-z)
Supplement: Supplementary file 1 — Appendix 1. (DOCX 20 kb) [file 13584_2019_339_MOESM1_ESM.docx]

Appendix 1. Pilot Study Methods and Results

This study was performed in two stages: Stage 1 was a retrospective analysis of screening provided by HACO as a service to the community via MCHC and preschools. Stage 2 was a prospective study of a second cohort of children that studied parental compliance with an invitation for vision screening at MCHC. The cohorts of the two stages do not overlap but involved children from the same target population.

*Ethics*: Stage 1: The Hadassah Academic College Ethics Committee granted permission for this retrospective, anonymous study of the findings from vision screenings by HACO, which was performed as part of student training and community service.

Stage 2: The Ministry of Health Helsinki Committee granted permission for Ministry of Health personnel to record percentage of compliance.

Preschool parents from stage 1 had received consent forms for their children to participate in the screening exam and only children with signed consent were examined. Parents from stage 2 consented to the procedure by phone and by bringing children in for screening.

*Target population*: Children between the ages of 3-5 residing in the Jerusalem area attending preschools or MCHC. The population of Israel is approximately 8.5 million with 1.2 million living in the greater Jerusalem area. The number of live births in Israel increases annually and there were 183,648 live births in 201, which is the most recent published data.

Study location for stages 1 and 2: MCHC in Israel provide free pediatric preventive health services, such as immunizations and neuro-developmental surveillance, separately from curative health care. MCHC provide care from birth to six years of age. Children are accompanied by a parent or guardian to all visits. The MCHC in our study were all operated by the Jerusalem District Health Office.

Study location for stage 1 only: The preschools studied were run by the Jerusalem Municipality and were part of Israel's Ministry of Education public preschool system for 3-5 year olds. Both MCHC and preschools provide services to children from various ethnic backgrounds and socioeconomic strata, including children who are native speakers of Hebrew, Arabic and Yiddish.

Study Protocol

Appointments: MCHC appointments for stages 1 and 2 were scheduled in advance via phone. In stage 2 of the project, the phone calls followed a set script. The MCHC staff explained the importance of vision screening, recorded the number of phone calls and appointments made. When parents refused an appointment, the refusal reason was recorded and fell into one of four categories: the child already had an eye exam with an ophthalmologist or optometrist, the hours available for appointments were inconvenient, the parent was not interested or other. The number of children who presented at the scheduled appointment was recorded.

*Screening exam*: The screening exam was part of the clinical training of optometry students at HACO. The screening team was equipped with all required instruments. Fourth and first year optometry students under the supervision of a licensed optometrist administered a battery of tests. Only the fourth year students, who have been trained in performing retinoscopy on children, performed this test. The tests performed were as followed. :

1. Monocular Distance Visual Acuity (VA) assessment with Lea Symbols with crowding bars at three-meter distance
2. Retinoscopy
3. Binocular vision testing
   1. Near Cover Test (1)
   2. Near Point of Convergence (NPC) and ocular motility
   3. Stereoacuity (depth perception, stereofly or stereosmile)

Any child who failed a test carried out by a student was re-examined on all vision tests by the supervising optometrist. The supervising optometrist also repeated all near cover tests and NPC exams. Table 2 lists the referral criteria. It should be noted that the optometrist sometimes referred children with borderline results based on her clinical experience.

Table 2. Referral Criteria for Pediatric Screening.

| Test | Referral criteria |
| --- | --- |
| Distance VA | 6/12 or worse * |
| Retinoscopy | Sphere >-1.0 D or >+1.5D, Cyl >1.5 D * |
| Cover test | Esophoria or tropia (squint) |
| Ocular Motility | Not equal, proper and full * |
| NPC | 10/12 or less |
| Stereopsis | Lower than 100 SOA |

*Child was referred if unable to cooperate with this test

VA – visual acuity; NPC – near point of convergence

*Data analysis:*

Descriptive statistics were used. Continuous variables such as referral rates were presented as proportions with 95% confidence intervals (CI's). Associations between the results of the clinical tests (passed/failed) with screening locations (preschools vs. MCHC) were analyzed by Chi-squared test. A two-sided *P-*value of 0.05 was considered statistically significant for all tests. Statistical analyses were performed using *SPSS* 23.0 software (Chicago, IL 60606-63070(.

**Results**:

At most locations, four simultaneous screening stations were created using portable screening kits. A team of HACO students ran each station. The supervising optometrist rotated through the stations. Each complete screening procedure took approximately 20-30 minutes, including re-examination by the optometrist. When possible, communication with children was in their native language: Hebrew, Arabic or Yiddish.

From Dec. 2016 to June. 2018, vision screening was carried out at 24 MCHCs and 40 preschools. The service was provided to 1476 children. The children were between 3-5 years of age, although the exact age, gender and the results of each procedure were not recorded as part of the stage 1 retrospective project. In stage 2 the mean age was 4.3 ± 0.4.

The prevalence of children referred for failing each examination in the screening protocol is shown in Table 3. Note that children may have been referred for failing more than one exam, so the sum of children failing each exam is larger than the overall failure rate. Like previous studies, more children failed in the VA and/or retinoscopy exams than tests for binocular vision. There was no statistically significant difference in the overall referral rate between preschools and MCHCs. Nor was there a difference in the referral rate for retinoscopy or binocular vision.

Table 3. Prevalence of Referral by Portion of Screening

|  | Total | MCHC | Preschool | P (χ²) |
| --- | --- | --- | --- | --- |
| Number of Children Examined | 1476 | 581 | 873 | 0.33 |
| Total referred N (%) | 353 (24%) | 124 (21%) | 224 (26%) | 0.22 |
| Referred for Non-cooperation N (%) | 52 (4%) | 24 (4%) | 28 (3%) |  |
| Referred for failure in Ret/VA N (%) | 217 (15%) | 70 (12%) | 145 (17%) | 0.19 |
| Referred for Failure in Binocular N (%) | 103 (7%) | 38 (7%) | 65 (7%) | 0.32 |

Compliance was measured in stage 2 of this project on a different cohort of children from the same MCHC district. Altogether, 1249 parents were contacted, of whom 66% scheduled appointments for their children at MCHC. The main reason parents gave for not scheduling an appointment was the inconvenient hours (42.9%), followed by the child having already had an eye exam (37.6%), not interested (15.1%) and other. Of the children who had scheduled study appointments, 310 (48.5%) presented at the MCHCs, giving an overall screening rate of 24.8%.

1. Vision in Preschoolers Study G. Does assessing eye alignment along with refractive error or visual acuity increase sensitivity for detection of strabismus in preschool vision screening? Invest Ophthalmol Vis Sci. 2007;48(7):3115-25.
